# ComplexMatrixComb: predicting anticancer drug combination dosages for target growth inhibition via complex-number matrix factorization

**DOI:** 10.1186/s13321-026-01261-y

**Published:** 2026-07-25

**Authors:** Mohammad Abdollahi, Shokoofeh Ghiam, Asiyeh Mirzaei Koli, Changiz Eslahchi

**Affiliations:** 1 School of Biological Sciences, Institute for Research in Fundamental Sciences (IPM), Farmanieh Campus, Tehran, 19395-5746 Iran; 2 Data and Computer Sciences, School of Mathematics, Shahid Beheshti University, Evin, Tehran, 1983969411 Iran

**Keywords:** Drug combination therapy, IC_50_ prediction, Dose-response modeling, Validation of the MTT assay, Matrix factorization

## Abstract

Determining precise drug concentration combinations required to inhibit cancer cell growth remains a critical yet resource-intensive challenge in oncology, particularly in combination therapies where exhaustive experimental screening of dose pairs is impractical. While most existing computational approaches focus on predicting drug synergy or classifying interaction types given predefined doses, they rarely address the inverse problem of estimating the specific drug concentration pairs needed to achieve a predefined inhibitory effect. In this study, we formally define and address the problem of drug combination dose estimation for a target level of growth inhibition, with a particular focus on achieving approximately 50% inhibition, a widely used and clinically relevant benchmark. We introduce ComplexMatrixComb, a novel complex-valued matrix factorization framework that models drug pair-cell line interactions by encoding the concentration of each drug as the real or imaginary component of a complex number. This representation enables the model to capture joint dose-response dynamics and predict drug concentration pairs that achieve the desired inhibitory effect within partially observed interaction spaces. We evaluated ComplexMatrixComb across three benchmark datasets (O’Neil, NCI-ALMANAC, and AZ-DREAM), where it consistently outperformed traditional machine learning models in both regression and classification tasks. The framework demonstrated robustness across heterogeneous experimental settings, stability under drug-order perturbations, and strong agreement between predicted and observed inhibition levels when evaluated alongside established inhibition prediction models such as ComboFM, ComboLTR and PanThera. To assess practical relevance, we experimentally validated five high-confidence drug pair-cell line predictions using MTT assays on previously untested combinations. The results confirmed that the model-predicted dose combinations induced inhibition levels close to the targeted 50% growth reduction. By directly addressing the challenge of drug combination dose estimation, ComplexMatrixComb reduces reliance on exhaustive experimental screening and provides a scalable, data-driven tool for preclinical drug combination design, with potential applications in precision oncology.

**Supplementary Information:**

The online version contains supplementary material available at https://doi.org/10.1186/s13321-026-01261-y.

## Scientific contribution

This study introduces the inverse drug combination dose prediction problem, which aims to estimate drug concentration pairs required to achieve a predefined inhibition level rather than predicting inhibition or synergy from predefined doses. We propose ComplexMatrixComb, a novel complex-valued matrix factorization framework that represents paired drug concentrations as complex numbers, enabling direct prediction of target-level dose combinations while capturing partner-dependent drug interactions. Experimental validation on previously untested drug pair-cell line combinations demonstrates the practical utility of the proposed approach for reducing exhaustive dose screening in preclinical drug combination studies.

**Supplementary Information:**

The online version contains supplementary material available at https://doi.org/10.1186/s13321-026-01261-y.

## Introduction

Cancer remains one of the leading health challenges worldwide, with millions of new cases diagnosed every year and high mortality rates in diverse populations. Numerous biological, genetic, and environmental factors contribute to the development and progression of different types of cancer   [1–5]. In recent years, bioinformatics has emerged as a crucial discipline to manage the complexity of cancer research. It supports various aspects of drug discovery and development, including protein targeting studies, next-generation sequencing analysis, functional annotation of genes and proteins, and identification of therapeutic biomarkers   [6–9]. The development of effective cancer therapies remains a major challenge in oncology, due to factors such as drug resistance, tumor heterogeneity, and the limited efficacy of single agents. One of the most widely used metrics for assessing drug potency is the half-maximal inhibitory concentration (IC_50_), which represents the concentration of a drug required to inhibit a biological or biochemical function by 50% [10]. Accurate estimation of IC_50_ plays a central role in drug discovery and pharmacology, as it enables researchers to evaluate and compare the potency of different substances in inhibiting cancer cell growth. Recent advancements in IC_50_ prediction highlight the growing application of advanced machine learning and computational techniques to enhance precision in drug response modeling. Accurate prediction of IC_50_ values is a central task in drug discovery and drug response modeling, and has been widely studied using a variety of computational paradigms. Existing approaches can be broadly grouped into several categories.

Feature-driven machine learning and deep learning models leverage molecular descriptors, gene expression, and multi-omics data to predict IC_50_ values for individual drugs across cell lines. These include graph neural networks, transformer-based architectures, and hybrid deep learning frameworks integrating chemical structure and transcriptomic information [11–14].

A second line of work formulates drug response prediction as a collaborative filtering or recommender-system problem, using matrix factorization and latent-variable models to infer missing entries in drug–cell line response matrices [15–17].

In parallel, structure-based and physics-informed strategies, including QSAR, SBDD, and quantum or DFT-based modeling approaches, have been explored to improve IC_50_ prediction accuracy and interpretability [18, 19].

While assessing the potency of single drugs using metrics like IC_50_ is critical for understanding their therapeutic potential, cancer treatment often requires more than a single-agent approach. Drug combinations have emerged as a promising strategy to overcome challenges such as drug resistance, tumor heterogeneity, and limited efficacy of individual agents. By leveraging the complementary mechanisms of action between drugs, combination therapies aim to enhance treatment outcomes, reduce toxicity, and minimize the likelihood of resistance [20–22]. The study of drug combinations has focused on several key problems, including predicting drug synergy scores, classifying drug interactions, and predicting inhibition levels. Each of these problems addresses a different aspect of understanding and optimizing drug combinations. Drug synergy occurs when the combined effect of two drugs is greater than the sum of their individual effects. Conversely, drug antagonism happens when the combined effect is less than the sum of their individual effects, leading to a reduced therapeutic outcome. Additivity refers to a situation where the combined effect of the drugs is exactly the sum of their individual effects, implying no interaction [23, 24]. From a computational perspective, methods for modeling drug combinations are typically formulated either as regression or classification problems. Regression-based approaches aim to predict continuous outcomes such as synergy scores, inhibition levels, or dose–response surfaces for drug pairs across cell lines. These methods include machine learning, deep learning, and matrix-factorization-based models that estimate quantitative measures of drug interaction from partially observed response data. Representative deep learning models such as DeepSynergy [25], DeepDDS [26], and MARSY [27] integrate chemical structure and gene expression information to predict quantitative synergy outcomes. Other machine learning and hybrid frameworks, including gradient boosting–based approaches and multi-feature deep architectures (e.g., DrugComb, DFFNDDS), similarly aim to predict continuous drug–drug interaction effects from multi-omics and structural data [28, 29]. These models primarily address forward prediction of quantitative interaction measures across drug pairs and cell lines.

In contrast, classification-based approaches seek to categorize drug pairs into discrete interaction types, such as synergistic, antagonistic, or additive combinations. Tools and models such as SynergyFinder [30], SynToxProfiler [31], MultiSyn [32], DEML [33], and SYNDEEP [34] use machine learning and deep learning architectures to identify interaction classes and predict synergy-related outcomes. These approaches are particularly useful for interaction discovery and prioritization; however, they typically focus on forward prediction of interaction strength or class and do not directly estimate dose combinations associated with a predefined inhibition level.

In practical therapeutic settings, a central question is how a given pair of drug doses affects cancer cell growth. That is, for a fixed cell line and a chosen combination of drug concentrations, what level of inhibition will be observed? This forward dose–response prediction problem has been extensively studied. Several computational frameworks, including comboFM [35], comboLTR [36], ComboKR [37], and ComboKR 2.0 [38], have demonstrated strong performance in modeling inhibition surfaces over predefined dose–dose grids. Similarly, probabilistic approaches such as PIICM [39] aim to predict dose–response landscapes while accounting for experimental uncertainty. These models estimate the inhibition effect associated with specified drug concentrations and are therefore well suited for forward prediction tasks.

However, from a decision-making perspective, an equally important question arises: for a given drug pair and a specific cancer cell line, which concentrations should be selected to achieve a desired inhibition level? This formulation corresponds to an inverse dose prediction problem. Rather than predicting the inhibition level at predefined doses, the objective is to infer dose combinations that produce a target response, such as IC_50_-level inhibition or, more generally, a specified inhibition threshold. Conceptually, this setting can be viewed as a natural extension of the classical single-drug IC_50_ problem, where the goal is to identify the concentration required to achieve 50% inhibition; here, the objective is to determine the pair of concentrations that jointly attain a target inhibition level for a specific cell line.

To address this challenge, we present *ComplexMatrixComb*, a matrix factorization framework designed to estimate the concentrations of two drugs required to achieve a predefined level of growth inhibition in a specific cancer cell line. Throughout this study, we focus on IC_50_-level inhibition (50% growth suppression) as a biologically meaningful and widely used reference point in pharmacology. However, the proposed approach is not restricted to IC_50_. ComplexMatrixComb is formulated to predict dose pairs corresponding to arbitrary target inhibition levels (IC_x_), enabling the identification of concentration combinations that achieve any desired inhibition threshold. This flexibility allows the framework to support diverse experimental and therapeutic objectives, including dose selection for follow-up assays and context-specific inhibition targets. Within this matrix factorization setting, responses of drug–pair and cell-line combinations are represented through latent embeddings that capture underlying interaction structure. A key modeling decision is how to represent drugs and their combinations in this latent space. Because the effective behavior of a drug depends strongly on its partner in a combination, assigning a single latent representation to each individual drug may not adequately capture its behavior across different interaction contexts. Such a formulation would implicitly assume that a drug’s latent effect remains consistent regardless of its combination partner, an assumption that often fails in practice due to context-dependent and non-additive interaction effects. To address this limitation, we treat drug pairs as atomic interaction units and learn pair-specific latent representations using a complex-valued matrix factorization formulation. In this formulation, the concentrations of the two drugs are encoded as the real and imaginary components of a complex number, enabling a compact and expressive representation of joint dose–response dynamics while preserving the independence of individual drug doses. This complex-valued structure allows the model to capture partner-dependent interaction effects and supports stable inverse mappings from target inhibition levels to dose combinations. To the best of our knowledge, this work is the first to explicitly formulate inverse dose prediction for drug combinations as a primary learning objective, rather than deriving effective dose pairs indirectly from forward response or synergy prediction models. As a result, ComplexMatrixComb provides a practical and scalable framework for directly identifying drug dose combinations that achieve predefined inhibitory effects, supporting more actionable and efficient combination therapy design. Importantly, the goal of the present study is not to reconstruct the full inhibition surface or the entire IC50 contour, but to identify a representative and actionable dose pair associated with a predefined inhibition target.

Solving the inverse dose prediction problem requires datasets that provide paired dose–response measurements for drug combinations across cell lines. In particular, the task relies on observations in which the concentrations of two drugs are jointly specified and the resulting inhibition level is measured for a given cell line. Such data allow the model to learn the relationship between dose pairs and inhibition outcomes and to infer concentration combinations associated with a target response level. Importantly, this setting does not require fully observed or perfectly dense dose–response grids; rather, it requires sufficiently structured measurements linking pairs of drug concentrations to observed inhibition levels. Several large-scale drug combination resources, including DrugComb and SYNERGxDB, aggregate data from multiple studies and serve as valuable benchmarks for synergy analysis. However, their primary focus is on reporting summary synergy scores or heterogeneously sampled dose–response measurements. In many cases, these resources do not provide consistently structured observations of paired drug concentrations and corresponding inhibition levels for specific drug–cell line combinations, which limits their suitability for inverse dose prediction. For this reason, we restrict our analysis to three widely used benchmark datasets that provide experimentally measured dose–response observations for drug pairs across cancer cell lines: the O’Neil (Merck) dataset [40], the NCI-ALMANAC dataset [41], and the AstraZeneca–DREAM dataset [42]. These datasets contain sufficiently structured dose–response measurements linking drug concentration pairs to inhibition outcomes, making them well suited to the inverse dose prediction setting considered in this study. We evaluate ComplexMatrixComb on the three datasets introduced above to assess its stability across heterogeneous experimental designs, its robustness to variations in drug ordering, and its performance relative to established forward modeling approaches such as ComboFM and ComboLTR. To assess practical applicability, we further performed experimental validation of selected high-confidence predictions using MTT assays. For the tested drug pair–cell line combinations, we examined whether the predicted dose pairs produced inhibition levels close to the intended IC_50_ target.

The remainder of this paper is organized as follows. We first describe the proposed methodology and the datasets used in our experiments. We then introduce the evaluation metrics and present a comprehensive performance analysis. Finally, we report experimental validation results, discuss the limitations of the current approach, and outline directions for future research.

## Method & materials

### Dataset

In this study, we conducted all analyses and evaluations using three widely used drug combination datasets: the O’Neil dataset, the NCI-ALMANAC dataset, and the AstraZeneca-DREAM (AZ-DREAM) challenge dataset. These datasets differ substantially in scale, experimental design, and biological coverage, allowing us to assess the robustness and stability of our method across diverse drug combination settings.

1. O’Neil dataset: We first used the O’Neil dataset produced by Merck & co. The original dataset contains 38 unique drugs; however, one drug lacked usable concentration–response measurements and was therefore excluded from our analysis. As a result, this dataset comprises 37 unique drugs forming 583 distinct drug pairs, each tested across 39 human cancer cell lines derived from seven tissue types. In total, the dataset contains 368,832 samples, where each sample corresponds to a specific drug pair, cell line, and measured inhibition level under a particular combination of drug concentrations.
2. NCI-ALMANAC dataset: The second dataset is derived from the NCI-ALMANAC, the largest publicly available drug combination screening resource. The full dataset includes more than 5000 combinations of approximately 100 FDA-approved small-molecule drugs screened against the NCI-60 panel of human tumor cell lines spanning nine tissue types, with over three million response measurements across multiple concentration pairs. Following the sampling strategy used in ComboFM to improve computational efficiency, we constructed a representative subset by randomly selecting 50 drugs (Supplementary Table S3) while preserving the original distribution of combination responses. Drug pairs with complete measurements across all relevant cell lines were retained, resulting in a refined dataset comprising 617 drug combinations of 50 unique drugs evaluated across 45 concentration pairs, yielding a total of 333,180 response measurements.
3. AstraZeneca-DREAM (AZ-DREAM) dataset: The third dataset used in this study is the AstraZeneca-DREAM challenge dataset, a widely adopted benchmark for drug combination analysis. This dataset includes experimental measurements for 91 unique drugs tested in 11,576 distinct drug combinations across 85 human cancer cell lines derived from diverse tissue types. Each drug combination is characterized by inhibition levels measured at multiple concentration pairs, providing comprehensive coverage of drug interaction effects. Due to its scale and diversity, the AZ-DREAM dataset is frequently used to evaluate drug synergy prediction methods.

It is worth emphasizing that these three datasets, O’Neil, NCI-ALMANAC, and AstraZeneca-DREAM, are the only publicly available resources that provide detailed information on drug combinations, including the inhibition levels of cell lines under various concentration pairs of two drugs, along with comprehensive coverage of tissue types.

### Method

In the datasets analyzed in this study, dose–response measurements are provided on predefined non-zero concentration grids and do not include explicit zero-dose boundary entries. Importantly, the proposed matrix factorization framework does not require fully observed dose–response matrices. During model evaluation, cross-validation explicitly introduces missing entries by withholding subsets of dose–response measurements, resulting in incomplete matrices during training. Missing or unsampled dose pairs are treated as unobserved entries during factorization, enabling the model to learn from partial screening data and ensuring compatibility with sparse and non-rectangular experimental designs. It is important to distinguish between the experimental dose–response matrices and the matrix used in the proposed factorization framework. Dose–response matrices describe the inhibition landscape of a specific drug pair on a given cell line and may be symmetric or non-symmetric, depending on the experimental design. ComplexMatrixComb does not impose any assumptions on the shape or symmetry of these experimental matrices. Instead, the model operates on a cell line by drug-pair matrix constructed from the available dose–response information. Since this matrix is inherently rectangular and non-symmetric, the proposed framework naturally supports both symmetric and non-symmetric experimental dose designs without any modification.

**Fig. 1:**
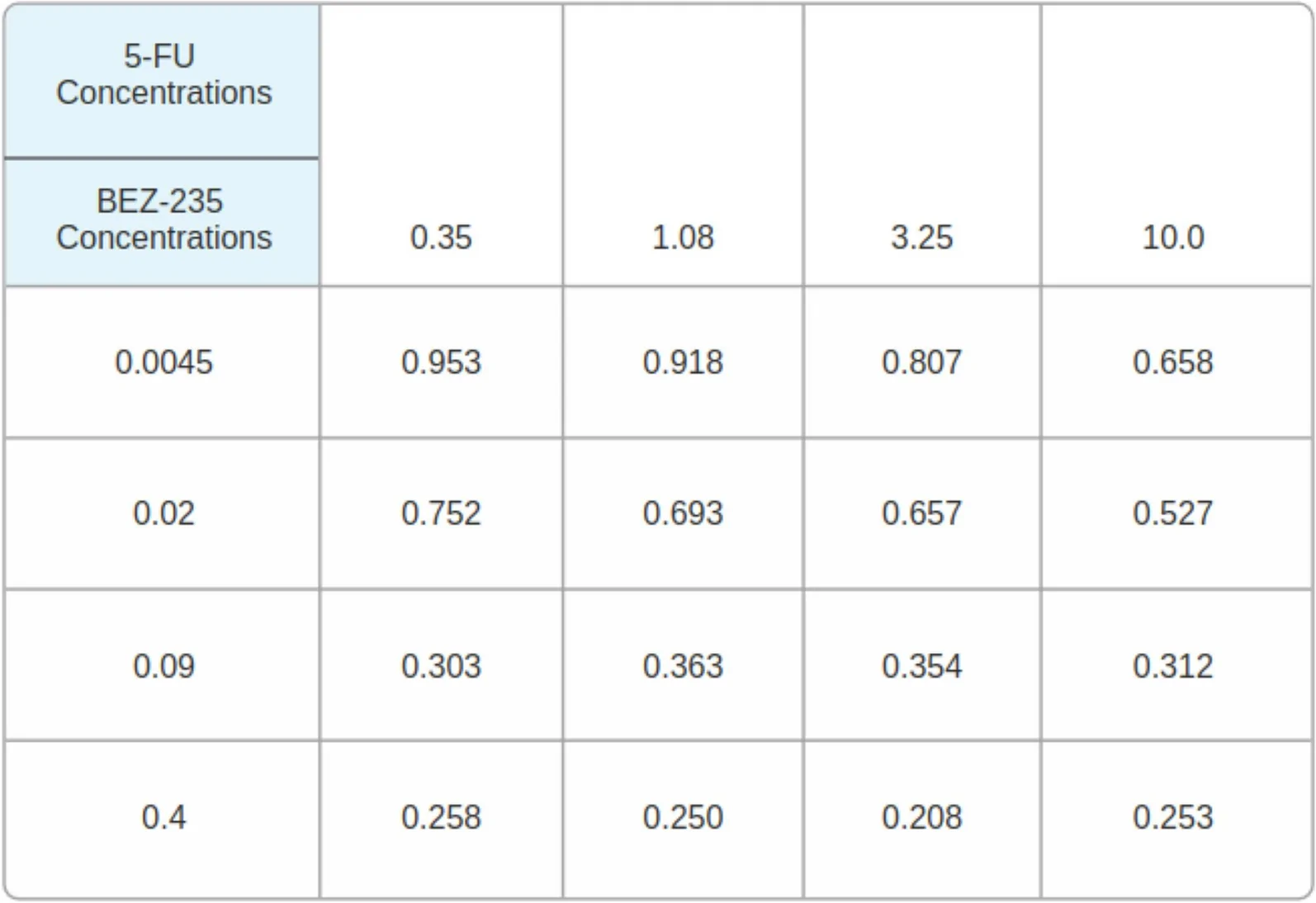
This figure illustrates a record from the O’Neil dataset, showing inhibition levels for the drug pair (5-FU, BEZ-235) applied to the A2058 cell line. The matrix represents drug concentrations in the micromolar (μM) scale, with rows corresponding to 5-FU concentrations and columns to BEZ-235 concentrations

Suppose $D = \{d_1, d_2,\ldots , d_n\}$ and $C = \{c_1, c_2,\ldots , c_m\}$ are the drugs and cell lines set in the dataset, respectively. Besides, for each pair of drugs $(d_i, d_j)$ and cell line c, there is a r×r drug-response matrix whose entries show the cell line inhibition level after applying different drug concentrations of these drug pairs. For instance, considering the drug pair (5-FU, BEZ-235) and the A2058 cell line from the O’Neil dataset, the data can be organized into a 4×4 matrix. The rows represent increasing concentrations of 5-FU, while the columns correspond to increasing concentrations of BEZ-235, with all drug concentrations reported in the micromolar (μM) scale. Each cell in the matrix reflects the observed inhibition level for the respective combination of concentrations of A2058 (see Fig. 1).

The main goal in ComplexMatrixComb is to implement an algorithm that predicts drug concentrations ${\mu }_{i}$ and ${\mu }_{j}$ for a pair of drugs such as $d_i$ and $d_j$ that with these concentrations in a specific cell line, leads to inhibition level close to 50 percent (between 45% and 55%).

The following are the steps of ComplexMatrixComb:

1. Scaling drug concentrations
2. IC_50_ inhibition level selection set
3. Create cell line-drug matrix with complex entries
4. Matrix factorization method
5. Estimate target-level dose pairs for observed drug pair–cell line combinations with missing target responses

**Fig. 2:**
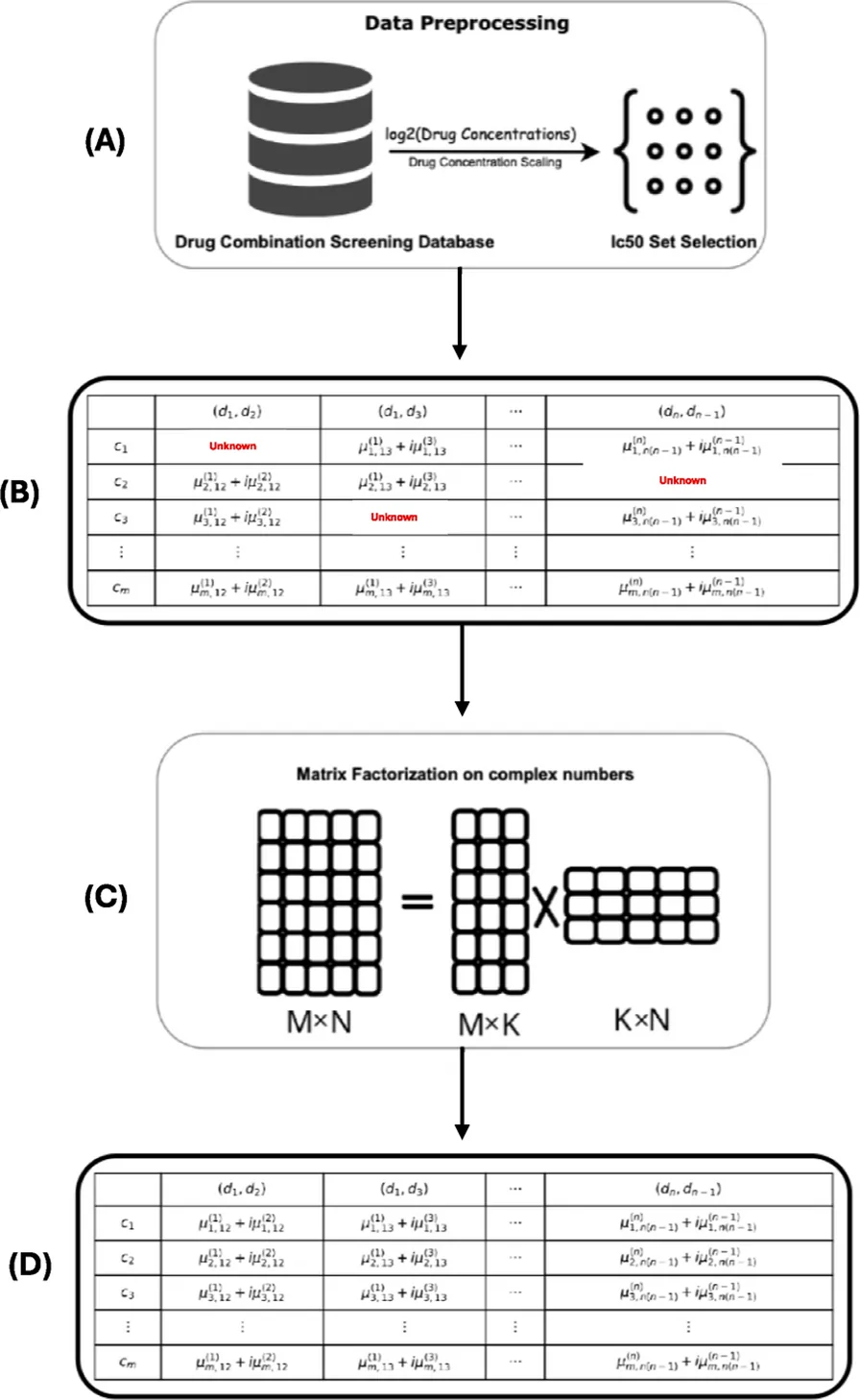
Schematic representation of the ComplexMatrixComb workflow. The pipeline includes preprocessing drug concentration matrices (**A**), selecting IC_50_ inhibition levels (**B**), constructing a complex-valued drug-cell line matrix (**C**), applying modified matrix factorization, and predicting effective drug pair concentrations that yield approximately 50% inhibition for specific cell lines (**D**)

In the following, the steps of the method are explained in detail and Fig. 2 demonstrates the workflow of our method.

#### Step1: scaling drug concentrations

For each pair of drugs $(d_i,d_j)$ and cell line c, there is a r×r matrix whose elements show the cell line inhibition level after applying different drug concentrations of these drug pairs. The matrix size is 4×4 for the O’Neil dataset, 3×3 for the NCI-ALMANAC dataset, and 5×5 for the AZ-DREAM dataset. To ensure the convergence of our algorithm, it is essential to address the variability in drug concentration values. A wide range of concentration values can hinder the convergence of our algorithm, making it challenging to achieve consistent and reliable results. To mitigate this issue, we will transform the drug concentration values by taking the logarithm base 2 ($log_2$) of each value.

By doing so, we achieve a more normalized distribution of values, which facilitates better convergence and stability in our computational methods.

#### Step2: IC_50_ inhibition level set selection

Suppose S denotes the IC_50_ inhibition level selection set, defined as follows: $S=\{{(\mu _{d_i}, \mu _{d_j}, r(c_k))|~ 45 \le r(c_k) \le 55,~ i,j \le n,~ k \le m}\}$ where $\mu _{d_i}$ and $\mu _{d_j}$ represent the concentrations for drugs $d_i$ and $d_j$, respectively, and $r(c_k)$ denotes the inhibition level for cell line $c_k$ after applying the drug pair ($d_i$, $d_j$) at these concentrations. This target-level selection follows directly from the learning objective rather than from a general preprocessing filter: because ComplexMatrixComb is formulated for inverse dose prediction at a predefined inhibition level, only dose pairs within the target inhibition window are used as direct supervision for the factorization model.

#### Step3: creating drug-cell line matrix with complex entries

**Fig. 3:**
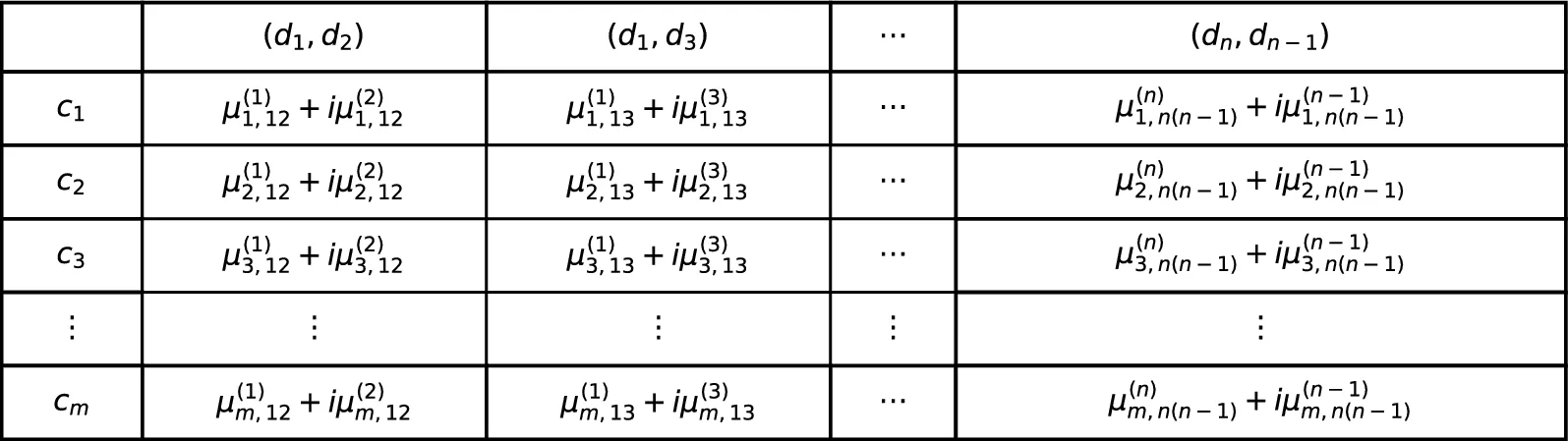
Each entry corresponds to a specific cell line–drug-pair combination. For a given cell line $c_k$ and drug pair $(d_i,d_j)$ with i<j, $\mu ^{(i)}_{k,ij}$ denotes the concentration-dose rate of drug $d_i$ when administered together with drug $d_j$ on cell line $c_k$, and $\mu ^{(j)}_{k,ij}$ denotes the corresponding dose of drug $d_j$ in the same pair and cell line. This indexing allows each drug to have a distinct rate depending on both its partner drug and the cell line

In this step, we construct a matrix $M = [m_{k, (i,j)}]$ corresponding to the set S. Each entry $m_{k, (i,j)}$ represents the value for column k, corresponding to cell line $c_k$ and row (i,j) corresponding to the drug pair ($d_i$, $d_j$). This entry is defined as: $m_{k, (i,j)} = \mu _{d_i} + i*\mu _{d_j}$, where $\{(\mu _{d_i}, \mu _{d_j}, r(c_k))\} \in S$. It is important to note that IC_50_ -level inhibition measurements are not available for every drug pair–cell line combination. This does not indicate missing experimental entries, but rather reflects cases in which none of the experimentally tested dose combinations for a given drug pair produce an inhibition level within the IC_50_ range (typically defined as 45–55% growth inhibition). As a result, the corresponding entries in *M* is missing value. We remove columns (representing drug pairs) that contain more than 50% missing values. Nevertheless, some missing entries still remain in the resulting matrix $C_0D_0$. These missing values were never treated as real observations: in our implementation, they were masked during training and therefore did not contribute to the optimization process. For technical reasons in the released code, these missing entries appear as the placeholder value 0+0i; however, this representation was used solely as a marker and was ignored during model fitting. Thus, the model was trained exclusively on valid concentration–response data without introducing any artificial zero-valued samples. The schematic representation of this matrix is shown in Fig. 3.

#### Step 4: matrix factorization method

Let $C_0D_0 \in \mathbb {C}^{m \times t}$ be the complex-valued matrix representing the interaction between m cell lines and t drug pairs. Our goal is to approximate $C_0D_0$ using low-rank matrix factorization, i.e.,

1 $$\begin{aligned} C_0D_0 \approx C D^T + B \end{aligned}$$

where:

- $C \in \mathbb {C}^{m \times h}$ is the complex-valued latent embedding matrix for cell lines,
- $D \in \mathbb {C}^{t \times h}$ is the complex-valued latent embedding matrix for drug pairs,
- $B \in \mathbb {C}^{m \times t}$ is a bias matrix,
- $h \ll \min (m, t)$ is the dimensionality of the latent space.

The approximation is optimized by minimizing the following loss function based on the $L_2$ norm:

2 $$\begin{aligned} \mathcal {L}(C, D, B) = \sum _{(i,j) \in \Omega } \left| {C_0D_0}_{i,j} - \left( \langle C_{i,:}, D_{j,:} \rangle + B_{i,j} \right) \right| \end{aligned}$$

where Ω denotes the set of observed (non-zero) entries, and $\langle \cdot , \cdot \rangle$ denotes the complex inner product. Each matrix entry is approximated as:

3 $$\begin{aligned} \hat{CD}_{i,j} = C_{i,:} \cdot D_{j,:}^T + B_{i,j} \end{aligned}$$

To optimize C, D, and B, we employ gradient descent using Wirtinger derivatives, which allow for differentiating real-valued loss functions with respect to complex variables. Specifically, for any complex variable z=x+iy, the gradients are computed as:

4 $$\begin{aligned} \frac{\partial \mathcal {L}}{\partial \Re (z)} = \Re \left( \frac{\partial \mathcal {L}}{\partial z} \right) , \quad \frac{\partial \mathcal {L}}{\partial \Im (z)} = \Im \left( \frac{\partial \mathcal {L}}{\partial z} \right) \end{aligned}$$

Consequently, the left-hand side of Eq. (4) updates the real component of the complex parameter, while the right-hand side updates its imaginary component. This formulation ensures that the real and imaginary parts are optimized jointly but independently during gradient-based learning. The parameters are updated iteratively as follows:

5 $$\begin{aligned} C_{i,:}&\leftarrow C_{i,:} - \alpha \left( \frac{\partial \mathcal {L}}{\partial \Re (C_{i,:})} + i \cdot \frac{\partial \mathcal {L}}{\partial \Im (C_{i,:})} \right) \end{aligned}$$

6 $$\begin{aligned} D_{j,:}&\leftarrow D_{j,:} - \alpha \left( \frac{\partial \mathcal {L}}{\partial \Re (D_{j,:})} + i \cdot \frac{\partial \mathcal {L}}{\partial \Im (D_{j,:})} \right) \end{aligned}$$

7 $$\begin{aligned} B_{i,j}&\leftarrow B_{i,j} - \alpha \left( \frac{\partial \mathcal {L}}{\partial \Re (B_{i,j})} + i \cdot \frac{\partial \mathcal {L}}{\partial \Im (B_{i,j})} \right) \end{aligned}$$

We also include an $L_2$ regularization term on the latent matrices to avoid overfitting:

8 $$\begin{aligned} \mathcal {L}_{\text {total}} = \mathcal {L} + \beta \left( \Vert C\Vert _F^2 + \Vert D\Vert _F^2 + \Vert B\Vert _F^2 \right) \end{aligned}$$

The algorithm is trained for a fixed number of epochs or until convergence.

This approach extends traditional matrix factorization methods  [43, 44] into the complex domain. We adopt the Wirtinger calculus framework  [45] to ensure valid and stable optimization for complex-valued parameters.

#### Step5: predict concentration values for *c*,  *d*, and $d^{\prime }$

After training the matrix factorization model, we obtain an embedding vector $$C_c$$ for each cell line c and an embedding vector $$D_{(d, d')}$$ for each drug pair $\left(d,d^{\prime }\right)$. Let the concentration values of the drug pair $\left(d,d^{\prime }\right)$ that inhibit the growth rate of cell line c by 50% are initially unknown. To estimate these concentration values, we calculate the dot product of $$C_c$$ and $$D_{(d, d')}$$. Following this calculation, we add the bias term $$B_{(c, (d,d'))}$$, where *B* is the bias matrix obtained in Step 4 of the method. The resulting complex number represents the $$log_2$$-transformed concentration value due to the normalization applied to the dataset. To retrieve the actual concentration values, we exponentiate the result using base 2. The resulting complex number is in the form $$\mu _{d} + i \cdot \mu _{d'}$$, where $$\mu _d$$ represents the concentration of the first drug d and $$\mu _{d'}$$ represents the concentration of the second drug $d^{\prime }$. In the current formulation, these predictions are restricted to drug pairs and cell lines represented in the training interaction matrix.

#### Implementation details

The model was trained for a maximum of 500 epochs or until convergence, defined as a relative change in the training loss of less than $10^{-5}$. Between consecutive epochs (see Table 1). To mitigate the risk of poor convergence and local optima inherent in the complex, non-convex optimization landscape, model parameters were initialized using Complex Xavier initialization [Glorot & Bengio, 2010], which promote stable gradient flow in the complex domain. In an addition $L_2$ regularization term (Equation 8) was incorporated to smooth the objective landscape and reduce overfitting. Hyperparameters listed in Table 1 were selected through empirical tuning on the O’Neil dataset to balance convergence stability and predictive accuracy. The hidden dimension *h* controls model capacity, while the regulation coefficient β further stabilizes optimization. Stochastic gradient decent (SGD) with learning rate α was employed to ensure steady loss reduction without oscillatory behavior. While an explicit multi-start strategy was not used, the chosen initialization and regularization scheme yielded stable and reproducible result across all cross validation folds (Section 3). Once tuned, this conformation was applied without modification to the ALMANAC and AZ-DREAM datasets, demonstrating its robustness across diverse experimental settings.

**Table 1:** Summary of implementation details

Hyperparameter	Value/Description
Hidden dimension size *h*	50
Initialization	Complex Xavier initialization
Optimizer	Stochastic Gradient Descent (SGD)
Learning rate α	0.01
Regularization coefficient β	0.001
Max number of epochs	500
Early stopping criteria	Relative loss change $< 10^{-5}$
Random seed	Fixed across all runs for reproducibility

## Results

In this section, we evaluated our method on three publicly available datasets: O’Neil, NCI60-ALMANAC, and AZ-Dream. To ensure robust and reliable evaluation, we applied 5-fold cross-validation on all datasets. In 5-fold cross-validation, the dataset is divided into five equal-sized subsets (or folds). Each fold is used as a testing set exactly once, while the remaining four folds are used as the training set. The process is repeated five times, with each fold serving as the test set in turn. The final performance metrics are obtained by averaging the results across all five iterations, providing a comprehensive assessment of performance stability across held-out folds.

This approach helps mitigate overfitting and reduces variability in the results by ensuring that all data points are used for both training and testing. Moreover, it provides insights into the stability and robustness of the method across different partitions of the data. Our evaluation consisted of two tasks regression and classification tasks:

- **Regression Task**: This task aimed to predict the optimal dosages of drug pairs that inhibit 50% of cancer cell line growth. Predicted dosages were compared with the ground truth values provided in the datasets, and the accuracy was measured using the Mean Squared Error (MSE) and Mean Absolute Error (MAE). For complex-valued predictions, these metrics are defined as:
  9 $$\begin{aligned} \text {MSE} = \frac{1}{N} \sum _{i=1}^N \left[ \left( \Re (\hat{y}_i) - \Re (y_i) \right) ^2 + \left( \Im (\hat{y}_i) - \Im (y_i) \right) ^2 \right] , \end{aligned}$$
  10 $$\begin{aligned} \text {MAE} = \frac{1}{N} \sum _{i=1}^N \left[ \left| \Re (\hat{y}_i) - \Re (y_i) \right| + \left| \Im (\hat{y}_i) - \Im (y_i) \right| \right] , \end{aligned}$$
  where $\Re (\cdot )$ and $\Im (\cdot )$ represent the real and imaginary parts of the predicted ($\hat{y}_i$) and actual ($y_i$) complex values, respectively.
- **Classification Task**: Because the benchmark datasets provide inhibition measurements on discrete concentration grids rather than continuous response surfaces, the target IC50 region is observed as a finite subset of experimentally tested dose combinations. Accordingly, the inverse prediction task considered here is formulated as identifying one representative target-level dose pair rather than reconstructing the full continuous contour. To provide an additional and intuitive evaluation of model performance, we reformulated the inverse dose prediction problem as a classification task. This formulation assesses whether the model can correctly identify, among a finite set of experimentally tested dose combinations, a drug concentration pair that achieves approximately 50% growth inhibition. In the benchmark datasets, each drug pair–cell line combination is associated with a discrete dose–response matrix obtained from multi-dose experiments. For example, in the O’Neil dataset, each drug–cell line pair is represented by a 4×4 matrix, corresponding to four predefined concentrations for each drug and resulting in 16 possible dose combinations with known inhibition values. As illustrated in Figure 1 for the O”Neil dataset, the drug pair (5-FU, BEZ-235) applied to the A2058 cell line yields a 4×4 dose–response matrix in which only one specific dose pair produces an inhibition level within the target IC_50_-inhibition level range (approximately 52%, i.e., within 45%–55% inhibition). If a dose pair were selected uniformly at random from the 16 possible matrix entries, the probability of choosing this IC_50_-inhibition level would be 1/16. This value therefore defines the baseline probability for the classification task in the O’Neil dataset. For the other benchmark datasets, the baseline probability is defined analogously as the inverse of the total number of dose combinations determined by the corresponding dose–response matrix size. Given a continuous dose pair predicted by the regression model, each drug dose is mapped to its nearest available concentration in the corresponding dose–response matrix. This mapping yields a single candidate dose pair among the experimentally tested combinations. A prediction is considered a true positive if the selected dose pair produces an inhibition level between 45% and 55%. Then classification accuracy is computed as,
  11 $$\begin{aligned} \text {Accuracy} = \frac{\text {TP}}{\text {Total number of evaluated predictions}} \end{aligned}$$
  where TP (True Positives) denote the number of predicted concentration pairs that, after being mapped to their nearest neighbors, inhibit cancer cell line growth between 45% and 55%. Accuracies substantially above the corresponding baseline indicate that the model effectively identifies dose combinations to the IC_50_ inhibition level obtained rather than selecting them by chance. To further complement the classification evaluation, we also introduced a continuous target-level deviation metric that measures how closely each predicted dose pair achieves the intended IC_50_ inhibition level. While the classification task evaluates whether a predicted dose pair falls within the 45%–55% target window, this additional metric quantifies the absolute deviation of the associated inhibition value from the target inhibition level of 50%. Formally, the deviation from the target inhibition level, $Dev_{50}$, is defined as
  12 $$\begin{aligned} Dev_{50} = \left| I_{obs}\left( NN(\hat{\mu }_1,\hat{\mu }_2)\right) - 50 \right| \end{aligned}$$
  where $I_{\textrm{obs}}$ denotes the experimentally observed inhibition value, $(\hat{\mu }_1,\hat{\mu }_2)$ is the predicted dose pair, and $\textrm{NN}(\cdot )$ denotes the nearest experimentally tested concentration pair in the corresponding dose–response matrix. Lower values indicate better agreement with the intended IC_50_ inhibition target.

**Table 2:** Performance metrics for regression task across datasets

Dataset	MAE (Log)	MSE (Log)	MAE (Original)	MSE (Original)
O’Neil	2.21±0.02	6.27±0.13	9.95±0.58	35.99±1.34
Almanac	3.07±0.003	12.26±0.20	12.56±0.16	23.25±0.37
AZ-Dream	2.67±0.05	9.51±0.32	1.38±0.18	6.12±1.024

**Table 3:** Performance metrics for the classification task and target-inhibition deviation across datasets

Metric	O’Neil	ALMANAC	AZ-Dream
Accuracy (%)	30.46±1.09	38.6±1.12	20.8±1.07
${Dev}_{{50}}$	10.21	11.53	19.74

Dev_50_ denotes the absolute deviation between the observed inhibition level at the predicted dose pair and the target inhibition level of 50%

### Results on the O’Neil dataset

- **Regression Results**: We performed 5-fold cross-validation on 2,106 test data points per fold to predict the optimal concentrations of drug pairs for 50% growth inhibition. The regression results, summarized in Table 2, highlight the model’s predictive performance. After applying log normalization, concentration values were constrained to the range (− 15, 9). The worst-case MSE occurs when the true concentration pair (9,9) is predicted as $(-\,15, -\,15)$, yielding an MSE of 1250 and an MAE of $24\sqrt{2}$. In contrast, across the 5 cross-validation folds, our model achieved significantly lower average MSE and MAE values of 6.27±0.13, and 2.21±0.02 respectively, where the values after the ± symbol represent the standard deviations. When converted back to the original scale, the average MSE and MAE were 35.99± 1.34 and 9.95± 0.58, both remaining far below the worst-case values. These results validate the model’s effectiveness in accurately predicting drug pair concentrations.
- **Classification Results**: We evaluated classification accuracy by measuring the proportion of correctly predicted drug-response pairs within the target inhibition range of (45–55%). Our model achieved an accuracy of 30.46±1.09, significantly surpassing the baseline random selection probability of at most (1/16) (Table 3). In addition, using the deviation-from-target metric, ComplexMatrixComb achieved a Dev_50_ value of (10.21), indicating the average absolute deviation between the observed inhibition level at the predicted dose pair and the target inhibition level of (50%).

### Results on the ALMANAC dataset

- **Regression Results**: We applied the same approaches used for the O’Neil dataset to the NCI-ALMANAC dataset. Specifically, we performed 5-fold cross-validation, with each fold containing 1,800 test data points. Unlike the O’Neil dataset, where the drug-response matrices were 4×4, the NCI-ALMANAC dataset uses 3×3 drug-response matrices. Each matrix represents 9 possible combinations of drug concentrations for a given drug pair and cell line. Log normalization constrained the concentration values to the range (− 18, 7). In the worst-case scenario, where the true concentration pair (7,7) is predicted as $(-\,18, -\,18)$,the maximum MSE is 2888, and the maximum MAE is $25\sqrt{2}$. In contrast, across the 5 cross-validation folds, our model demonstrated significantly lower errors, achieving an average normalized MSE of 12.26±0.20 and MAE of 3.07±0.003. When converted back to the original scale, the average MSE and MAE were 23.25±0.37 and 12.56±0.16, respectively. (Table 2)
- **Classification Results**: We applied a similar approach as used for the O’Neil dataset to evaluate classification performance on the NCI-ALMANAC dataset. Specifically, we transformed the regression task into a classification task by mapping the predicted continuous drug concentrations to their nearest neighbors within the constrained 3×3 drug-response matrix of the ALMANAC dataset. As described for the O’Neil dataset this results in a random guess having a baseline probability of at most 1/9 for correctly identifying an effective concentration pair. Our model achieved an accuracy of 38.6± 1.12 (Table 3), which is 3.6 times higher than the baseline probability. In addition, ComplexMatrixComb achieved a Dev_50_ value of 11.53, indicating that the observed inhibition levels associated with the predicted dose pairs remained close to the target inhibition level of (50%).

### Results on the AZ-Dream dataset

- **Regression Results**: We applied a similar approach as used for the O’Neil and NCI-ALMANAC datasets to the AZ-Dream dataset. Specifically, we performed 5-fold cross-validation, with each fold containing 1096 test data points, to predict the optimal concentrations of drug pairs for achieving 50% growth inhibition. The regression results are summarized in Table 2. After applying log normalization, concentration values were constrained to the range [-16,6]. In the worst-case scenario, where the true concentration pair (− 16, − 16) is predicted as (6,6), the maximum Mean Squared Error (MSE) and Mean Absolute Error (MAE) values were calculated as 968 and $22\sqrt{2}$, respectively. However, across the 5 cross-validation folds, our model demonstrated significantly lower average errors, achieving a normalized MSE of 9.51±0.19 and MAE of 2.67±0.04, where the values following the ± symbol represent standard deviations. When predictions were converted back to the original scale, the model exhibited similarly robust performance, achieving an average MSE of 6.12±0.27 and MAE of 1.38±0.08, both substantially below the worst-case scenario values.
- **Classification Results**: We also applied a similar classification approach as used for the O’Neil and NCI-ALMANAC datasets to evaluate the AZ-Dream dataset. The regression task was reformulated as a classification problem to identify drug-response pairs within the target inhibition range of $[45\%, 55\%]$. Predicted continuous concentrations were mapped to the nearest neighbors within the 5×5 drug-response grid of the AZ-Dream dataset. The baseline probability for random selection was at most 1/25, derived from the 5×5 grid structure. In contrast, our model achieved an accuracy of 0.20±0.01 (Table 3), which is five times higher than the baseline probability. In addition, ComplexMatrixComb achieved a Dev_50_ value of (19.74), indicating the average absolute deviation between the observed inhibition level at the predicted dose pair and the target inhibition level of (50%).

These results highlight the robustness and reliability of our approach in identifying effective drug pair concentrations that inhibit cell growth within the desired range, further demonstrating the robustness of the method across diverse datasets.

### Regression performance: inverse dose prediction accuracy

**Fig. 4:**
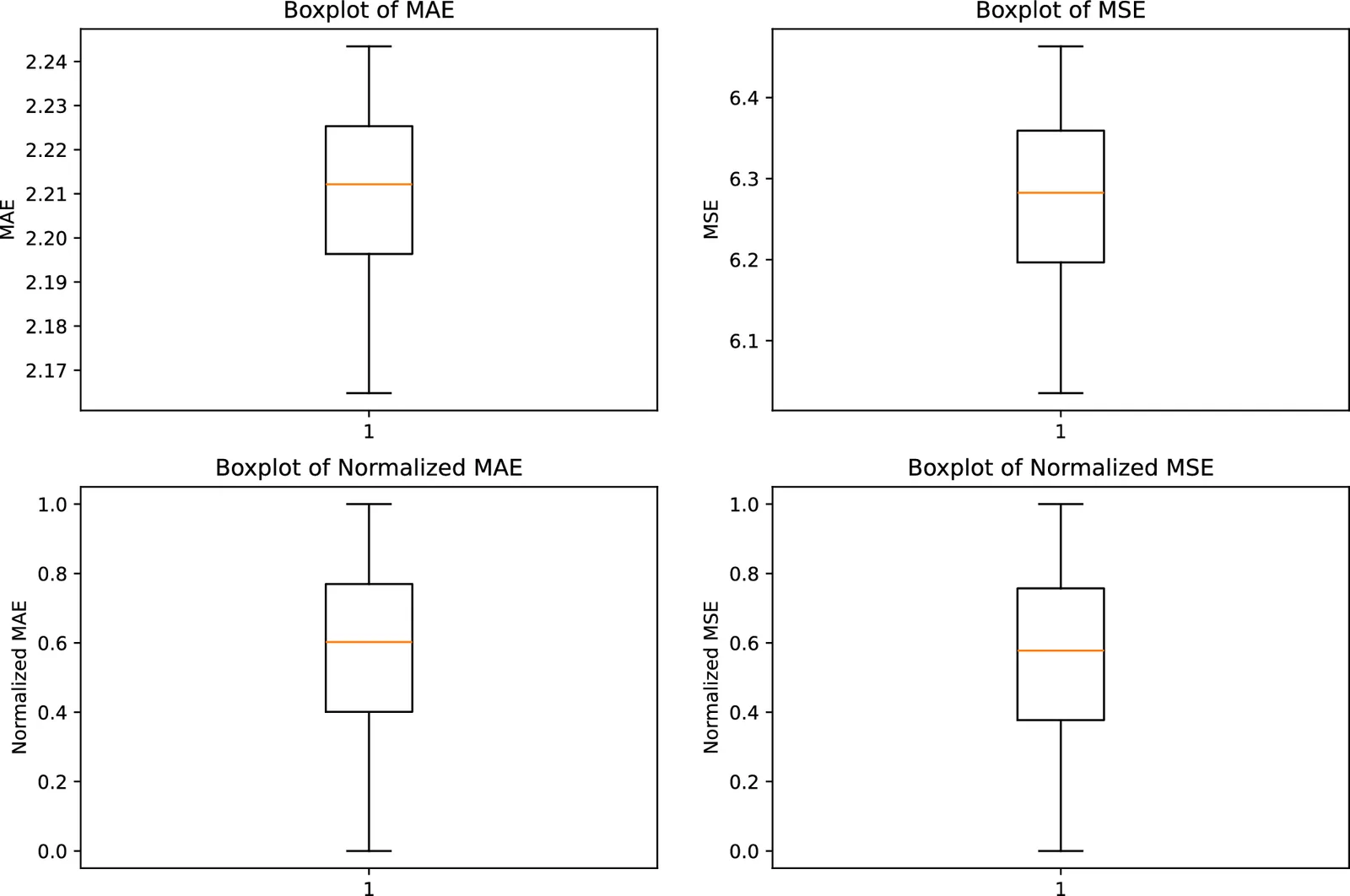
Boxplots of MAE, MSE, normalized MAE, and normalized MSE obtained from 30 independent repetitions of 5-fold cross-validation on the O’Neil dataset, illustrating performance variability and robustness of ComplexMatrixComb with respect to data partitioning

To assess the robustness of the proposed method with respect to data partitioning, we performed repeated cross-validation experiments. The standard 5-fold cross-validation procedure was repeated 30 times using independent random splits of the data. For each repetition, MAE and MSE, along with their normalized variants, were computed and summarized using boxplots. As shown in Fig. 4, both raw and normalized error metrics exhibit limited variability across repetitions on the O’Neil dataset, indicating stable performance and low sensitivity to data partitioning. This analysis complements the average error metrics reported above by providing an empirical characterization of performance variability. The same repeated cross-validation protocol was applied to the ALMANAC and AZ-DREAM datasets. In both cases, the distributions of raw and normalized error metrics show similarly limited variability, consistent with the observations on the O’Neil dataset (Supplementary Figs. S1 and S2).

**Fig. 5:**
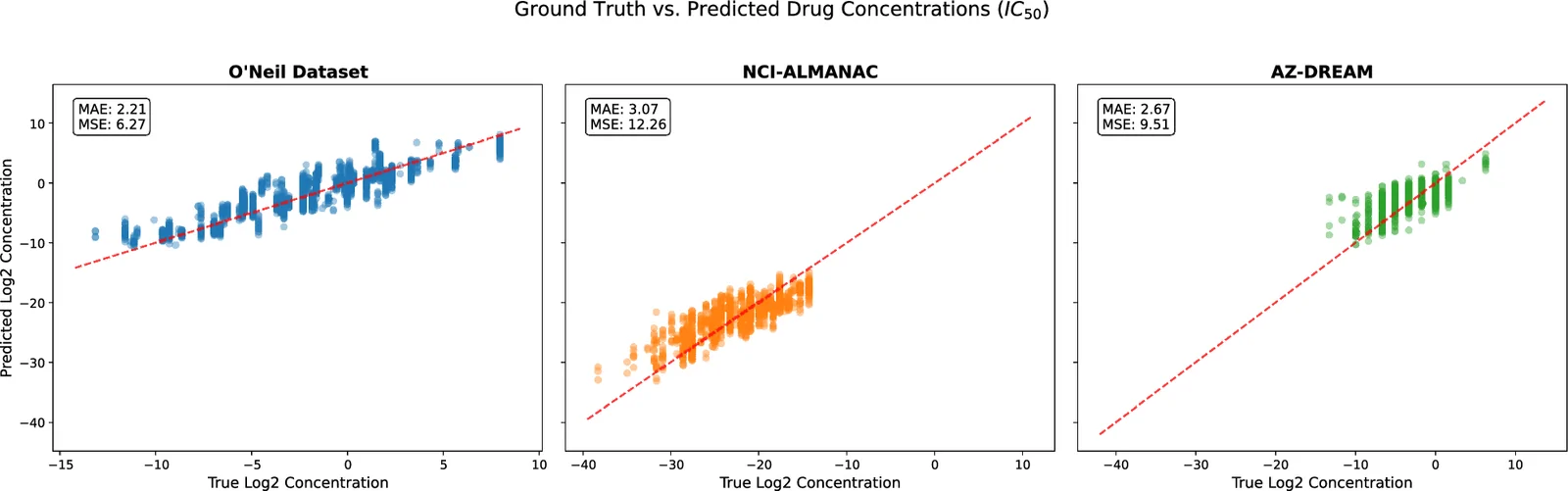
Scatter plots comparing ground-truth and predicted drug concentrations. For each dataset (O’Neil, NCI-ALMANAC, and AZ-DREAM), predicted log$_2$-transformed drug concentrations are plotted against the corresponding ground-truth values for a representative fold of the five-fold cross-validation. The dashed diagonal line indicates perfect agreement between predictions and true concentrations. These plots provide an instance-level view of inverse dose prediction accuracy, complementing aggregate error metrics by highlighting dispersion and potential systematic deviations across the tested concentration range

To provide an intuitive and instance-level assessment of inverse dose prediction accuracy, we visualized the regression performance of ComplexMatrixComb using scatter plots that compare predicted and ground-truth drug concentrations. Specifically, for each dataset (NCI-ALMANAC, O’Neil, and AstraZeneca), we report scatter plots from a representative fold of the five-fold cross-validation, where the x-axis corresponds to the true log2-transformed concentration and the y-axis to the predicted log2-transformed concentration, Fig. 5. These plots directly illustrate how closely the model’s predictions align with the ground-truth concentrations across the tested range and complement aggregate error metrics by highlighting systematic deviations and dispersion at the individual prediction level.

### Comparing complex matrix factorization with alternative machine learning methods

A key objective of this study is to assess the effectiveness of our matrix factorization-based approach compared to alternative machine learning methods. One possible alternative is to treat the problem as a multi-output regression task, where a model directly predicts the optimal drug concentrations, $\mu _i$ and $\mu _j$, based on a common set of input features. This approach differs from our complex matrix factorization method, which operates on a matrix with complex-valued entries. Instead of directly predicting drug concentrations, matrix factorization decomposes this complex-valued matrix to learn latent drug interaction patterns, enabling more accurate predictions within the evaluated transductive setting. To investigate this, we implemented a regression-based pipeline inspired by ComboFM. The data preprocessing steps remained consistent with ComboFM; however, instead of using factorization to infer drug interactions, we reformulated the problem as a supervised regression task. Here, the target outputs were the concentrations of $d_1$ and $d_2$, while the inhibition level was included as an additional feature.

We also evaluated the following baseline models:

- ElasticNet, a linear regression method with L1 and L2 regularization.
- Random Forest, an ensemble learning approach that builds multiple decision trees.
- Multilayer Perceptron (MLP), a four-layer neural network trained to predict drug concentrations.

For ElasticNet, we used a regularization ratio of 0.5 (equal weight for L1 and L2 penalties) and performed grid search over alpha values in the range $[10^{-4}, 10^{-1}]$. For the Random Forest model, we used 100 trees with a maximum depth of 20 and employed mean squared error (MSE) as the splitting criterion. Hyperparameters were tuned using 5-fold cross-validation on the training data. The MLP consisted of four fully connected layers with 128, 64, 32, and 16 hidden units, respectively, each followed by a ReLU activation. The model was trained using the Adam optimizer with a learning rate of 0.001 and early stopping based on validation loss. Dropout with a rate of 0.2 was applied to mitigate overfitting.

All methods, including our complex matrix factorization model, were tested on three independent datasets, O’Neil, ALMANAC, and AZ-Dream, under identical experimental conditions. We used the same 5-fold cross-validation strategy described earlier, ensuring that all models were trained and evaluated using the same data splits.

Table 4 presents MAE and MSE for each approach in the three datasets. The results clearly demonstrate that the complex matrix factorization-based method outperforms the alternative machine learning models, achieving consistently lower error rates. While the Random Forest model performed better than ElasticNet and MLP, it still lagged behind our method, particularly on the ALMANAC and AZ-Dream datasets. These findings suggest that performing matrix factorization on a complex-valued interaction matrix provides a more effective way to capture latent drug interactions, leading to more accurate dosage predictions than direct regression-based approaches.

**Table 4:** Performance comparison of our complex matrix factorization approach against alternative machine learning models (ElasticNet, Random Forest, and MLP) on three benchmark datasets (O’Neil, ALMANAC, and AZ-Dream)

Method	O’Neil		ALMANAC		AZ-Dream	
	MAE	MSE	MAE	MSE	MAE	MSE
ElasticNet	5.04±0.04	30.08±1.33	5.41±0.01	35.38±1.79	3.71±0.01	17.24±0.87
RandomForest	2.37±0.06	7.03±0.25	4.23±0.004	21.34±0.29	3.36±0.005	13.54±0.41
MLP	4.20±0.03	21.55±4.01	4.01±0.19	19.09±1.8	3.61±0.01	16.36±0.91
Matrix Factorization	2.21±0.02	6.27±0.13	3.07±0.003	12.26±0.20	2.67±0.05	9.51±0.32

Metrics reported are Mean Absolute Error (MAE) and Mean Squared Error (MSE), averaged over five-fold cross-validation

As an alternative model architectures we also considered the possibility of replacing matrix factorization component with more complex deep learning architectures, such as Transformer-based models and other attention-based or graph-based neural networks. However, the available data was not sufficient to effectively train these high-capacity models. Despite experimenting with several configurations, their performance was significantly worse than our factorization-based method due to overfitting and lack of convergence. As a result, we do not include these models in the main comparison table, since their poor results were largely due to data limitations rather than methodological inferiority. Instead, we focused on more stable and reproducible baselines that perform reliably under low-data regimes.

### Evaluating model predictions using ComboFM, ComboLTR and PanThera

To evaluate the performance of ComplexMatrixComb, we compared it against interpolation-based IC_50_-level dose estimation strategies derived from forward inhibition prediction models, namely ComboFM, ComboLTR and PanThera. In these baselines, the full dose-response observations in each training fold are used to learn inhibition surfaces over the dose–dose grid. We considered two evaluation scenarios.

In the first scenario (Real concentrations; RC), the predicted inhibition surface is queried directly at the experimentally observed ground-truth dose pair for each held-out test sample. A prediction is counted as correct if the inhibition value predicted at that dose pair falls within the 45–55% range. This scenario evaluates how well each forward model’s predicted surface is locally aligned with the true IC_50_ inhibition region when queried at experimentally measured concentrations. In the second scenario (Predicted concentrations; PC), after training ComboFM, ComboLTR and PanThera, we extracted an IC_50_-level contour from each predicted inhibition surface by intersecting the surface with the 50% inhibition plane. This scenario evaluates whether forward models can recover a target-level dose combination indirectly via surface prediction and interpolation. Because forward surface-prediction models may yield multiple candidate dose pairs along the predicted IC_50_-level contour, we did not reduce the contour to a single arbitrary grid point before computing regression error. Instead, for the regression evaluation, all IC_50_-level candidate dose pairs obtained from the predicted surface were considered. The dose-coordinate error was computed between each candidate dose pair and the held-out ground-truth IC_50_-level dose pair, and the minimum error among these candidates was reported. This best-candidate protocol was used to avoid unfairly penalizing forward surface-prediction methods solely because of sparse experimental grids or the existence of multiple valid IC_50_-level solutions. Therefore, the PC regression results for ComboFM, ComboLTR and PanThera should be interpreted as optimistic best-candidate errors for forward surface-prediction baselines, whereas ComplexMatrixComb directly outputs a single inverse dose-pair prediction.

For the classification evaluation of forward surface-prediction baselines, each candidate dose pair on the predicted IC_50_-level contour was independently mapped to the nearest available concentration pair in the corresponding experimental dose-response matrix. If at least one of these mapped grid points produced an observed inhibition value within the 45%–55% target range, the prediction was counted as correct. This best-candidate classification protocol gives forward surface-prediction methods the benefit of all IC_50_-level candidates identified from their predicted surface and avoids penalizing them for returning a contour rather than a single dose pair. Therefore, the reported classification accuracy for these baselines should be interpreted as an optimistic estimate under the PC setting. Using the same best-candidate protocol, we also computed Dev_50_ for these baselines. When multiple candidate dose pairs were available, the minimum Dev_50_ value among all candidates was reported.

The RC scenario represents an optimistic upper bound for forward inhibition models, because it bypasses the inverse step of recovering IC_50_-level dose candidates from the predicted surface and instead evaluates the predicted surface directly at the true dose location. As expected, accuracies under RC are generally higher than under PC. Accordingly, RC primarily serves as a diagnostic for assessing the local alignment of predicted inhibition surfaces with the true IC_50_ inhibition region, whereas PC reflects the more practically relevant inverse task of inferring IC_50_-level dose combinations from predicted inhibition surfaces alone. Table 5 summarizes the performance of ComboFM, ComboLTR and PanThera under both evaluation scenarios. As expected, the RC setting yields higher accuracies than the PC setting for all methods. A direct comparison between the classification results in PC setting denoted in Table 5 shows that ComplexMatrixComb outperforms all forward baselines on the O’Neil and NCI-ALMANAC datasets, while showing comparable performance on AZ-DREAM. Specifically, on the O’Neil dataset, ComplexMatrixComb achieves 30.46% ± 1.09 accuracy, compared with 30.1% ± 0.01 for ComboFM, 29.9% ± 0.01 for ComboLTR and 30.42% ± 0.52 for PanThera. On NCI-ALMANAC, the improvement is more pronounced, with ComplexMatrixComb reaching 38.6% ± 1.12 versus 24.9% ± 0.01, 20.6% ± 0.01, and 32.70% ± 1.32 for ComboFM, ComboLTR, and PanThera respectively. On AZ-DREAM, ComplexMatrixComb attains 20.8% ± 1.07, compared with 20.7% ± 0.05 for ComboFM, 21.7% ± 0.04 for ComboLTR and 21.75% ± 1.52 for PanThera. Table 6 reports Dev_50_, which measures the absolute deviation between the observed inhibition level associated with a predicted dose pair and the target inhibition level of 50%. Lower values indicate better agreement with the intended IC_50_ inhibition target. ComplexMatrixComb achieved the lowest Dev_50_ values on the O’Neil and AZ-DREAM datasets, obtaining values of 10.21 and 19.74, respectively. On the NCI-ALMANAC dataset, ComboFM achieved the lowest Dev_50_ value (9.09), while ComplexMatrixComb obtained a competitive value of 11.53. Overall, these results demonstrate that the dose pairs predicted by ComplexMatrixComb consistently achieve inhibition levels close to the desired IC_50_ target and remain competitive with state-of-the-art forward surface-prediction approaches, including ComboFM, ComboLTR, and PanThera.

Table 7 summarizes the regression performance of ComplexMatrixComb, ComboFM, ComboLTR, and PanThera under the PC scenario. Overall, ComplexMatrixComb achieves the lowest MAE and MSE on the O’Neil and NCI-ALMANAC datasets, indicating more accurate inverse dose prediction than the forward surface-prediction baselines in these settings. On O’Neil, ComplexMatrixComb attains an MAE of 2.21±0.02 and an MSE of 6.27±0.13, outperforming ComboFM, ComboLTR, and PanThera. A similar pattern is observed on NCI-ALMANAC, where ComplexMatrixComb yields the lowest MAE (3.07±0.003) and MSE (12.26±0.20), again surpassing all three forward baselines. On AZ-DREAM, ComplexMatrixComb remains competitive and improves over ComboFM and PanThera, although ComboLTR achieves the best regression performance on this dataset. These results indicate that direct inverse optimization in ComplexMatrixComb provides a generally effective strategy for target-level dose estimation compared with recovering dose candidates indirectly from predicted inhibition surfaces, with the clearest advantage observed on the O’Neil and NCI-ALMANAC datasets.

**Fig. 6:**
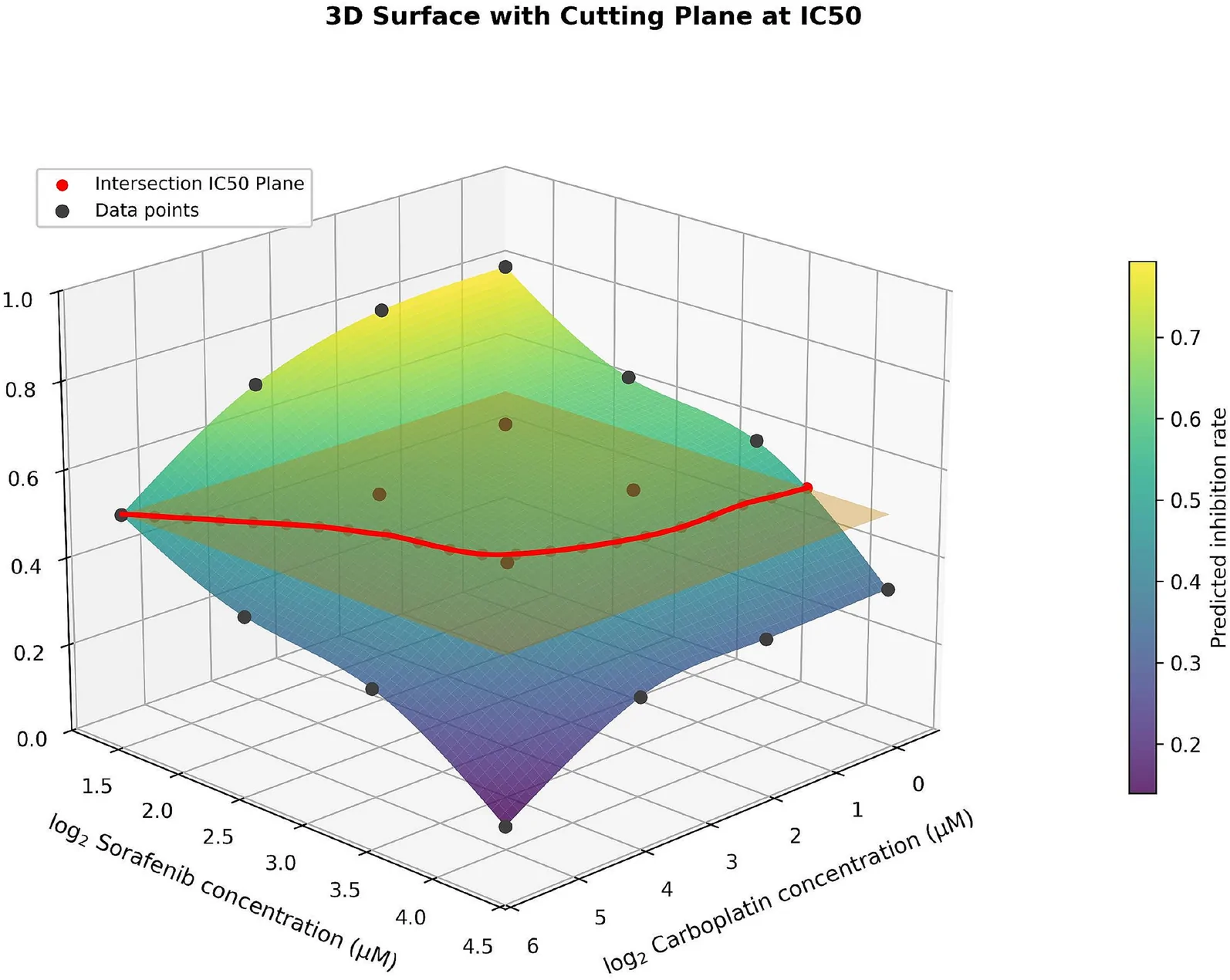
Predicted inhibition surface generated by ComboFM for the Carboplatin–Sorafenib combination on the A2058 cell line. The horizontal plane denotes the 50% inhibition level. The intersection between the predicted inhibition surface and this plane defines an IC_50_-level contour, which is subsequently projected onto the experimental concentration grid to obtain a single grid-based prediction under the evaluation protocol described in Section 3.6

To illustrate this effect concretely, we present a representative example using the drug pair Carboplatin–Sorafenib on the A2058 cell line. In this example, we trained ComboFM to predict the inhibition surface over the dose–dose grid and then identified the IC_50_-level contour by intersecting the predicted surface with the 50% inhibition plane and interpolating along the resulting intersection. The held-out ground-truth dose pair achieving approximately IC_50_-level inhibition in the dataset is $(d_1, d_2)=(50.0, 2.5)$, whereas the closest interpolation-derived estimate produced by ComboFM under the evaluation protocol is $(d'_1,d'_2)=(12.75, 10.0)$, which deviates substantially from the true target-level dose pair. This example highlights that deriving dose combinations achieving IC_50_-level inhibition from forward inhibition models requires an additional interpolation step and may accumulate error. In contrast, ComplexMatrixComb is explicitly trained to directly predict dose pairs corresponding to a predefined inhibition level as its primary learning objective, resulting in more accurate and stable inverse dose predictions (see Fig. 6).

**Fig. 7:**
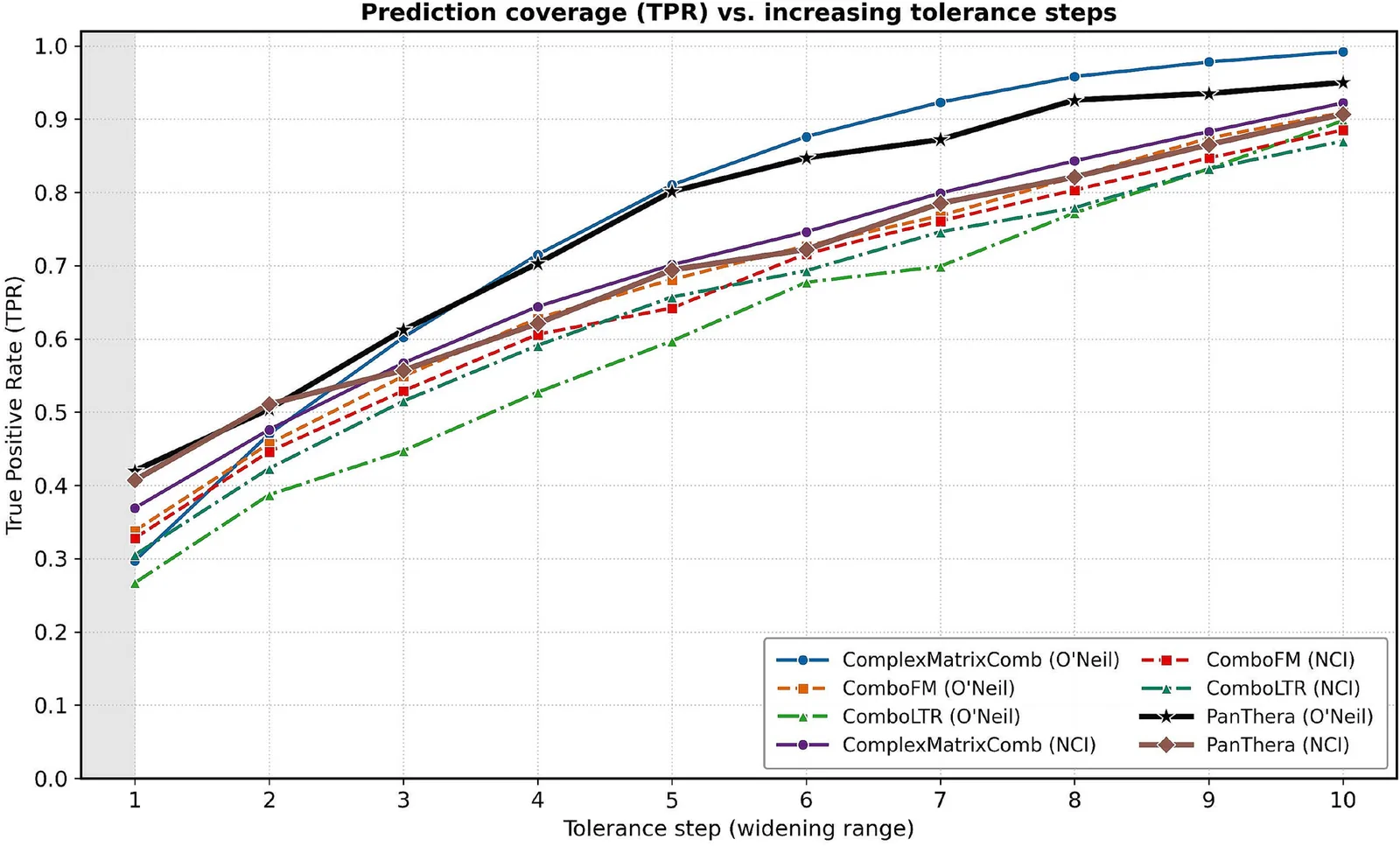
Coverage–tolerance curves for inverse IC_50_ inhibition dose prediction. The true positive rate (TPR) is plotted as a function of increasing tolerance thresholds around the IC_50_ inhibition level, providing a regression-aware, classification-style view of prediction accuracy. Curves are shown for ComplexMatrixComb and the baseline forward inhibition models, comboFM, comboLTR and PanThera on the O’Neil and NCI-ALMANAC datasets. Higher curves indicate greater prediction coverage at comparable tolerance levels, reflecting more reliable inverse dose prediction under relaxed accuracy constraints

While the primary evaluation above focuses on tolerance-based classification at a fixed IC_50_ inhibition level, inverse dose prediction is inherently a regression problem with continuous-valued outputs. To further characterize model behavior beyond a single tolerance window, we therefore introduce a regression-aware evaluation that retains an interpretable classification-style perspective. Specifically, we report coverage–tolerance curves that quantify prediction coverage as a function of increasing tolerance thresholds around the IC_50_ inhibition level. For each method, the true positive rate (TPR) is computed as the fraction of evaluated predictions whose inhibition values fall within a progressively widened tolerance range centered at 50% inhibition. By varying the tolerance window, these curves provide an intuitive visualization of how prediction accuracy evolves under relaxed precision constraints. This analysis complements the fixed-threshold classification results by illustrating model behavior across a continuum of tolerance levels rather than at a single operating point. The coverage–tolerance curves are reported for ComplexMatrixComb as well as the baseline forward inhibition models, comboFM, comboLTR, and PanThera enabling direct visual and quantitative comparison. As shown in Fig. 7, ComplexMatrixComb consistently attains higher coverage at comparable tolerance levels on the O’Neil and NCI-ALMANAC datasets. An analogous pattern is observed for the AZ-DREAM dataset, although these curves are omitted from the figure for clarity.

**Table 5:** Comparison of classification accuracy for ComboFM, ComboLTR, PanThera and ComplexMatrixComb (CMC) on the O’Neil, NCI-ALMANAC, and AZ-DREAM datasets

Method	O’Neil		ALMANAC		AZ-Dream	
	RC	PC	RC	PC	RC	PC
ComboFM	34.8±0.01	30.1±0.01	25.8±0.01	24.9±0.01	25.8±0.0001	20.7±0.05
ComboLTR	31.7±0.01	29.9±0.01	21.8±0.01	20.6±0.01	26.4±0.01	21.7±0.04
PanThera	35.99±1.48	30.42±0.52	37.62±1.30	32.70±1.32	26.94±1.12	$\mathbf {21.75\pm 1.52}$
CMC	N/A	$\mathbf {30.46\pm 1.09}$	N/A	$\mathbf {38.6\pm 1.12}$	N/A	20.8±1.07

RC and PC denote real-concentration and predicted-concentration settings, respectively. The RC setting is applicable only to forward surface-prediction baselines because it evaluates the predicted inhibition surface at experimentally observed concentration pairs. Since ComplexMatrixComb directly predicts target-level dose pairs and does not predict inhibition surfaces at externally supplied concentrations, RC is not applicable to this method

**Table 6:** Comparison of deviation from the target inhibition level (Dev_50_) for ComplexMatrixComb, ComboFM, ComboLTR, and PanThera across datasets

Method	O’Neil	ALMANAC	AZ-Dream
ComboFM	11.42	9.09	20.26
ComboLTR	27.02	22.38	27.62
PanThera	10.31	10.17	20.93
ComplexMatrixComb	10.21	11.53	19.74

Lower values indicate better agreement with the target inhibition level of 50%

**Table 7:** Regression performance of ComplexMatrixComb, ComboFM, ComboLTR, and PanThera under the predicted-concentration (PC) scenario on the O’Neil, NCI-ALMANAC, and AZ-DREAM datasets

Method	O’Neil		ALMANAC		AZ-Dream	
	MAE	MSE	MAE	MSE	MAE	MSE
ComboFM	2.45±0.02	6.86±0.33	3.31±0.02	13.01±0.39	2.75±0.01	9.87±0.17
ComboLTR	2.32±0.05	6.41±0.15	3.23±0.05	12.87±0.19	$\mathbf {2.34\pm 0.01}$	$\mathbf {9.38\pm 0.44}$
PanThera	3.15±0.04	10.81±0.28	3.61±0.06	18.54±0.24	3.59±0.06	12.85±0.42
ComplexMatrixComb	$\mathbf {2.21\pm 0.02}$	$\mathbf {6.27\pm 0.13}$	$\mathbf {3.07\pm 0.003}$	$\mathbf {12.26\pm 0.20}$	2.67±0.05	9.51±0.32

MAE and MSE values are averaged over five-fold cross-validation

### Robustness and invariance to drug pair ordering

In biological systems, the interaction between two drugs should not depend on their order in the dataset representation. To examine whether our framework is sensitive to this arbitrary ordering choice, we assessed its robustness to drug pair reordering. This property is important because, from a biological perspective, the designation of “first” and “second” drugs in a pair has no intrinsic meaning. To test this, we randomly swapped the order of the two drugs in 50% of the dataset and then retrained and evaluated the model using five-fold cross-validation on the O’Neil, ALMANAC, and AZ-Dream datasets. The same data splits were used for both the original and reordered versions to ensure a fair comparison. Under this experiment, swapping the real and imaginary components of the complex representation corresponds to exchanging the roles of Drug 1 and Drug 2 in a consistent manner across both training and testing, while leaving the underlying biological interaction unchanged. As summarized in Table 8, the model exhibited highly consistent performance across all key metrics, indicating strong empirical robustness to drug pair reordering. For example, on the O’Neil dataset, the MAE remained nearly identical between the original (2.21±0.02) and reordered (2.20±0.02) datasets, with accuracy also showing minimal variation (30.42±1.14 vs. 30.46±1.09). Similar trends were observed for ALMANAC and AZ-Dream, where the differences in MAE, MSE, and accuracy between the original and reordered datasets were negligible. These results show that the proposed framework is robust in practice to permutation of drug order when the reordered representation is used consistently throughout the learning pipeline. This should be interpreted as an empirical robustness property rather than as a strict claim that swapping the real and imaginary parts of a fixed trained model would leave its predictions unchanged. Overall, the observed stability across datasets further supports the reliability of our approach for modeling drug interactions in diverse settings.

**Table 8:** Performance metrics of the model on original and modified shuffled datasets for O’Neil, ALMANAC, and AZ-Dream

Dataset	Metric	Original data	Modified data
O’Neil	MAE	2.21±0.02	2.20±0.02
	MSE	6.27±0.13	6.27±0.12
	Accuracy	30.42±1.14	30.46±1.09
ALMANAC	MAE	3.07±0.003	3.06±0.003
	MSE	12.26±0.20	12.25±0.18
	Accuracy	38.6±1.12	38.7±0.008
AZ-Dream	MAE	2.67±0.05	2.67±0.06
	MSE	9.51±0.32	9.50±0.30
	Accuracy	20.8±1.07	20.9±1.05

### Experimental prediction and validation of drug pair doses using MTT assay

In drug combination research, experimental datasets often suffer from incomplete coverage due to the high cost and time required for preclinical testing. As a result, many drug pair–cell line combinations lack experimental measurements at specific inhibition thresholds, such as the IC_50_ inhibition level. These gaps hinder the systematic identification of effective drug combinations and limit progress in precision oncology. To address this challenge, we developed a two-phase strategy that integrates predictive modeling with experimental validation. In the first phase, the proposed model was trained on the complete set of available drug–response data to learn patterns of combination efficacy. The trained model was then used to impute missing IC_50_ inhibition level in drug–response matrices, focusing specifically on drug pair–cell line combinations for which IC_50_ inhibition measurements were unavailable. Model performance was evaluated using five-fold cross-validation on drug pair–cell line combinations with known IC_50_ inhibition level. For each drug pair, we assessed the agreement between predicted and experimentally measured values on held-out test folds. Drug pairs that consistently exhibited low prediction error and close alignment with ground-truth measurements across cross-validation folds were considered high-confidence predictions, reflecting robust generalization performance. In the second phase, these high-confidence drug pairs were prioritized for experimental validation on cell lines for which no IC_50_ inhibition measurements were available in the original datasets. The model was used to predict dose combinations expected to achieve approximately 50% growth inhibition, and these predicted doses were subsequently evaluated using MTT assays. This experimental design enabled direct assessment of whether drug pairs that generalized well on observed data also produced accurate predictions in genuinely unobserved experimental settings. Accordingly, the following drug pair–cell line combinations were selected from the ALMANAC and O’Neil datasets for experimental validation, based on their consistently strong predictive performance across cross-validation folds and the absence of experimentally measured IC_50_ inhibition level for the corresponding combinations.

- **From the ALMANAC dataset:**
  - Ifosfamide and Gefitinib on OVCAR-3
  - Ifosfamide and Gefitinib on HL-60
  - Ifosfamide and Gefitinib on ACHN
  - Ifosfamide and Gefitinib on HCT-116
- **From the O’Neil dataset:**
  - Gemcitabine and Sorafenib on HCT-116

#### Experimental validation using MTT assay

To test the validity of the predicted IC_50_ inhibition level, we conducted in vitro experiments using MTT assays on selected drug pair–cell line combinations. Four human cancer cell lines were selected for experimental validation:

- HCT-116 (colon cancer)
- OVCAR-3 (ovarian cancer)
- HL-60 (leukemia)
- ACHN (kidney cancer)

The details are demonstrated in Table 9.

All cell lines were obtained from the National Cell Bank of Iran. Cells were cultured in RPMI-1640 medium (Gibco), supplemented with 10% fetal bovine serum (FBS) and penicillin-streptomycin (100 U/mL and 100 µg/mL, respectively). Cultures were maintained at 37 °C in a humidified incubator with 5% CO_2_ and passaged using trypsin-EDTA.

Cell viability was assessed using the MTT assay (Sigma-Aldrich). Cells were seeded in 96-well plates at a density of $1.4 \times 10^4$ cells/well in 200 µL RPMI medium. After 24 h, they were treated with predicted IC_50_ inhibition drug combinations and incubated for an additional 24 h. Each condition was tested in technical triplicates and repeated across three independent biological replicates.

- Negative control (untreated)
- Vehicle control (DMSO-treated)

In this experiment, no separate cytotoxic positive control compound was included in the MTT assays, as the primary objective of the experiments was to validate whether the model-predicted drug dose combinations could achieve approximately 50% growth inhibition. The tested drug pairs themselves consist of cytotoxic agents with known anticancer activity and were evaluated directly against untreated and vehicle controls.

After treatment, the following *MTT* procedures apply:

1. Remove media and add 100 µL of MTT solution (0.5 mg/mL in PBS).
2. Incubate for 4 h at 37 °C.
3. Remove MTT solution and add 100 µL of DMSO.
4. Measure absorbance at 570 nm using an ELISA reader.

The average absorbance in untreated control wells was 0.704, representing 100% viability. All drug-treated conditions resulted in a marked reduction in absorbance, confirming the cytotoxic effects of the predicted doses and aligning closely with IC_50_ inhibition level expectations. The results were consistent across replicates. Slight variability observed in samples treated on OVCAR-3 and HL-60 may reflect cell line–specific sensitivities. No significant outliers were detected, indicating high reproducibility of the experimental protocol (Table 10).

These findings confirm that the predicted doses effectively induced approximately 50% reduction in cell viability, validating the model’s accuracy across multiple cancer cell lines and drug combinations. This outcome supports the broader utility of computational dose prediction in preclinical settings and underscores its potential to streamline experimental design in drug discovery and precision oncology.

**Table 9:** Predicted Drug Combinations and Applied Doses

Sample	Cell Line	Tissue Type	Drug 1	Dose 1 (mol)	Drug 2	Dose 2 (mol)
1	OVCAR-3	Ovary	Ifosfamide	$5.23 \times 10^{-6}$	Gefitinib	$5.51 \times 10^{-6}$
2	HL-60	Hematopoietic	Ifosfamide	$5.6 \times 10^{-6}$	Gefitinib	$4.65 \times 10^{-6}$
3	ACHN	Kidney	Ifosfamide	$1.18 \times 10^{-6}$	Gefitinib	$4.86 \times 10^{-6}$
4	HCT-116	Colon	Ifosfamide	$6.41 \times 10^{-9}$	Gefitinib	$5.36 \times 10^{-6}$
5	HCT-116	Colon	Gemcitabine	$4.259 \times 10^{-9}$	Sorafenib	$7.42 \times 10^{-6}$

**Table 10:** Observed Viability After Treatment

Sample	Cell Line	Drug Pair	Viability (%)	Standard Deviation (%)
1	OVCAR-3	Ifosfamide + Gefitinib	46	±10.05
2	HL-60	Ifosfamide + Gefitinib	51	±8.23
3	ACHN	Ifosfamide + Gefitinib	49	±2.78
4	HCT-116	Ifosfamide + Gefitinib	48	±1.56
5	HCT-116	Gemcitabine + Sorafenib	49	±1.04

## Discussion

Combination therapy has become a central paradigm in the treatment of complex diseases such as cancer, offering the potential to enhance therapeutic efficacy, mitigate drug resistance, and reduce toxicity through multi-target modulation. Despite substantial progress in computational modeling of drug combinations, most existing approaches address the forward problem, that is, predicting inhibition, synergy, or response for a given drug pair at specified concentrations. In contrast, the inverse problem of directly predicting the dose combinations required to achieve a predefined biological effect, such as an IC_50_-level response, has remained largely unexplored. This gap is particularly consequential from an experimental design perspective, where the efficient identification of effective dose combinations is often more valuable than reconstructing full dose-response landscapes. In this sense, forward surface-predicting models and direct inverse dose-prediction models address related but fundamentally different objectives: the former reconstruct inhibition landscapes, whereas the latter directly support dose selection for a predefined target inhibition level. In this work, we introduced ComplexMatrixComb, a complex-valued matrix factorization framework that formulates inverse dose prediction for drug combinations as a first-class learning objective. An important design choice in ComplexMatrixComb is to model drug pairs as atomic interaction units rather than constructing pair representations from embeddings of individual drugs. In combination dose–response settings, the effect of a drug is inherently dependent on the identity of its partner, and a single latent vector assigned to an individual drug may not generalize consistently across different pairing contexts. The latent representation of a drug that best explains its interaction with one partner may not adequately describe its interaction with another, particularly under non-additive and dose-dependent interaction regimes. By factorizing a matrix whose columns correspond to drug pairs and whose rows correspond to cell lines, the proposed framework learns pair-specific embeddings directly from observed combination responses. This avoids imposing compositional assumptions on how individual drug representations should combine and allows the model to capture pair-dependent dose–response landscapes more accurately in inverse prediction settings. We acknowledge that inductive formulations based on single-drug embeddings could enable prediction for entirely unseen drug pairs. Such extensions would require a transition to feature-driven or compositional architectures and represent an important direction for future work. The present study instead focuses on accurate inverse dose estimation within experimentally observed combination spaces, where pair-specific modeling provides a more appropriate representation. Across three heterogeneous benchmark datasets (O’Neil, NCI-ALMANAC, and AZ-DREAM), the proposed method demonstrated stable and consistent performance in both regression and classification settings. Repeated cross-validation experiments further confirmed the robustness of these results, with narrow interquartile ranges across 30 independent repetitions, indicating low sensitivity to data partitioning. Scatter plot visualizations of predicted versus ground-truth concentrations provided an instance-level assessment of inverse dose prediction accuracy, revealing close alignment along the identity line and limited systematic bias across the tested concentration ranges. To contextualize inverse dose prediction within a classification-oriented evaluation framework, we assessed whether predicted dose pairs achieved IC_50_ inhibition level within predefined tolerance ranges. ComplexMatrixComb consistently exceeded random baselines across all three datasets, highlighting its ability to identify biologically meaningful dose combinations under constrained experimental grids. ROC-style coverage–accuracy curves further demonstrated that, under progressively widened tolerance thresholds, ComplexMatrixComb maintains superior performance relative to baseline methods. This advantage is particularly pronounced on the NCI-ALMANAC dataset, where the benefits of direct inverse learning are most evident. A central contribution of this work is a controlled methodological comparison between direct inverse dose prediction and IC_50_-level dose estimation derived from forward inhibition-surface models. Although approaches such as comboFM and comboLTR predict full inhibition landscapes over dose–dose grids, identification of IC_50_-level combinations requires an additional interpolation step, introducing an extra layer of approximation. To isolate the core methodological contrast, we restrict our quantitative comparison to comboFM and comboLTR, which constitute representative forward-surface baselines. Extended variants such as ComboKR and ComboKR 2.0 introduce additional modeling components and are therefore left for future investigation. Across datasets, interpolation-based extraction of IC_50_ dose pairs consistently underperforms direct inverse prediction. This performance gap suggests that modeling the inverse mapping from desired inhibition level to dose combinations as a primary objective yields more reliable IC_50_-level identification than deriving it post hoc from predicted response surfaces. In this study, IC_50_ inhibition level is used as a canonical reference point for inverse dose prediction, owing to its widespread adoption in pharmacology and drug combination screening as a standardized measure of drug efficacy. Importantly, however, the proposed framework is not intrinsically limited to IC_50_ inhibition level. ComplexMatrixComb is formulated to predict dose pairs corresponding to an arbitrary predefined inhibition target, and the choice of 50% inhibition serves primarily as a consistent and interpretable reference across datasets. From a modeling standpoint, alternative inhibition levels (IC_x_, e.g., IC_30_, IC_70_, or IC_90_) can be accommodated naturally by adjusting the target inhibition criterion during training and evaluation, without modifying the underlying architecture. By treating the inhibition level as a controllable parameter rather than a fixed endpoint, the framework enables targeted inverse dose prediction tailored to specific experimental or therapeutic objectives. Beyond purely computational evaluation, we experimentally validated a subset of high-confidence predictions using MTT assays across multiple cancer cell lines. All tested drug pair–cell line combinations resulted in cell viability values close to 50%, providing strong empirical support for the biological plausibility and translational relevance of the predicted doses. This experimental confirmation reinforces the practical utility of ComplexMatrixComb for guiding targeted follow-up experiments and reducing the cost and scope of exhaustive preclinical screening. Despite ComplexMatrixComb strengths, the framework has several limitations. First, like many matrix factorization and collaborative filtering approaches, ComplexMatrixComb is transductive in nature and relies on partially observed drug–cell line interaction data. As a result, it is not explicitly designed to address fully cold-start scenarios involving entirely unseen drugs, unseen cell lines, or de novo drug pairs. Accordingly, the current formulation should be interpreted as a transductive inverse dose-prediction framework for partially observed interaction spaces, rather than an inductive model for unseen entities. This design choice aligns with common preclinical screening settings, where partial interaction information is typically available and the primary challenge lies in estimating effective dose combinations within known experimental contexts. Incorporating side information, such as drug chemical descriptors or cell line genomic profiles, represents a promising direction for extending the framework to more inductive settings. A second limitation arises from the structure of the available benchmark datasets. The drug combination data analyzed in this study are provided as constrained r × r dose–response matrices for each drug pair and cell line, where rows and columns correspond exclusively to nonzero concentrations of the two drugs. These matrices do not include explicit single-agent conditions (i.e., zero-dose rows or columns), which precludes direct estimation of classical monotherapy IC_50_ values. Consequently, direct comparison with monotherapy IC_50_–based baselines is not feasible within the current experimental setting. Future datasets that incorporate explicit monotherapy arms or zero-dose conditions would enable direct comparison between inverse combination dose prediction and classical single-agent IC_50_ modeling, representing an important avenue for future work. Finally, the proposed framework is intentionally designed to operate within experimentally validated concentration ranges, prioritizing biologically meaningful and actionable dose selection over unconstrained extrapolation. While this choice enhances practical relevance, extending the model to handle broader extrapolative regimes or explicitly address batch effects and cross-dataset generalization remains an open challenge. In summary, this work introduces a novel and practical framework for inverse dose prediction in drug combination studies. By directly learning dose pairs that achieve predefined inhibition levels, ComplexMatrixComb complements existing forward modeling approaches and provides a targeted tool for experimental prioritization. The demonstrated robustness across datasets, improved performance relative to interpolation-based baselines, and successful experimental validation collectively suggest that inverse dose prediction can play a valuable role in accelerating preclinical drug combination discovery and supporting precision oncology workflows.

## Conclusion

Identifying precise drug dose combinations that achieve a desired biological effect remains a central bottleneck in combinatorial cancer therapy design. While substantial progress has been made in modeling drug synergy and forward dose–response behavior, the inverse problem of directly predicting dose combinations that achieve a predefined inhibition level has largely remained unaddressed. In this work, we introduce ComplexMatrixComb, a complex-valued matrix factorization framework that, to the best of our knowledge, constitutes the first systematic approach to direct inverse dose prediction for drug combinations. By formulating inverse dose prediction as a primary learning objective and encoding paired drug concentrations within a complex-valued latent space, ComplexMatrixComb departs fundamentally from existing forward modeling paradigms. Rather than reconstructing full inhibition surfaces and deriving effective doses through post hoc interpolation, the proposed method directly learns mappings from target inhibition levels to actionable dose pairs. This formulation enables accurate and stable predictions within constrained screening grids, while remaining computationally efficient and well suited to low-data regimes commonly encountered in preclinical studies. Importantly, the proposed framework is not restricted to a single inhibition threshold. ComplexMatrixComb can be readily applied to predict dose combinations corresponding to arbitrary inhibition levels (IC_x_), supporting flexible experimental objectives ranging from low-toxicity screening to more aggressive inhibition strategies. By reducing reliance on exhaustive dose–response experiments, the method has the potential to accelerate preclinical screening, lower experimental costs, and improve prioritization of candidate drug combinations in precision oncology workflows. In summary, this work establishes inverse dose prediction as a distinct and practically relevant computational paradigm in drug combination research. By shifting the focus from forward response modeling to direct dose inference, ComplexMatrixComb complements existing methodologies and provides a foundation for more efficient, targeted, and biologically grounded combination therapy design.

## Supplementary Information

Supplementary material 1

## Data Availability

The source code and datasets are available at: https://github.com/mohammad−abdollahi/ComplexMatrixComb.
