# Supplementary material for: ComplexMatrixComb: predicting anticancer drug combination dosages for target growth inhibition via complex-number matrix factorization
Source: J Cheminform. 2026 Jul 25;18:147. doi: 10.1186/s13321-026-01261-y (PMC13693093; doi:10.1186/s13321-026-01261-y)
Supplement: Supplementary file 1 [file 13321_2026_1261_MOESM1_ESM.pdf]

Supplementary Data

Table S1: List of drug compounds in the selected subset of the NCI-ALMANAC dataset.

**Drug Name**	**Mechanism/Targets**	**Class**
2-Fluoro Ara-A	Antimetabolite; Purine analog	Chemotherapy
Amifostine	Antineoplastic detoxifying agent	Chemotherapy
Arsenic trioxide	Antineoplastic agent	Chemotherapy
Axitinib	VEGFR, PDGFR, KIT inhibitor	Targeted therapy
Bortezomib	Proteasome inhibitor (26S subunit)	Chemotherapy
Cabazitaxel	Taxane microtubule stabilizer, antimitotic	Chemotherapy
Carmustine	Alkylating agent	Chemotherapy
Chlorambucil	Nitrogen mustard alkylating agent	Chemotherapy
Cladribine	Antimetabolite; Purine analog	Chemotherapy
Crizotinib	ALK, c-Met inhibitor	Targeted therapy
Cytarabine hydrochloride	Antimetabolite, interferes with DNA synthesis	Chemotherapy
Dacarbazine	Alkylating agent	Chemotherapy
Dactinomycin	RNA and DNA synthesis inhibitor	Chemotherapy
Daunorubicin hydrochloride	Topoisomerase II inhibitor	Chemotherapy
Erlotinib hydrochloride	EGFR inhibitor	Targeted therapy
Estramustine phosphate sodium	Alkylating agent	Chemotherapy
Everolimus	binds FKBP12, causes inhibition of mTORC1	Targeted therapy
Exemestane	Aromatase inhibitor	Other
Floxuridine	Antimetabolite	Chemotherapy
Fulvestrant	Estrogen receptor antagonist	Other
Gefitinib	EGFR inhibitor	Targeted therapy
Hydroxyurea	Antineoplastic agent	Chemotherapy
Ifosfamide	Nitrogen mustard alkylating agent	Chemotherapy
Lenalidomide	Immunomodulatory	Other
Lomustine	Alkylating nitrosourea compound	Chemotherapy
Megestrol acetate	Progestogen	Other
Melphalan	Nitrogen mustard alkylating agent	Chemotherapy
Methoxsalen	Antineoplastic agent	Chemotherapy
Mitotane	Antineoplastic agent	Chemotherapy
Mitoxantrone	Topoisomerase II inhibitor	Chemotherapy
Oxaliplatin	Platinum-based antineoplastic agent	Chemotherapy
Pemetrexed Disodium	Dihydrofolate reductase inhibitor	Chemotherapy
Pralatrexate	Antifolate	Chemotherapy
Procarbazine hydrochloride	Alkylating agent	Chemotherapy
Quinacrine hydrochloride	Antineoplastic agent	Other
Romidepsin	HDAC inhibitor	Targeted therapy
Ruxolitinib	JAK1&2 inhibitor	Targeted therapy
Sorafenib tosylate	B-Raf, FGFR-1, VEGFR-2 & -3, PDGFR-beta, KIT, and FLT3 inhibitor	Targeted therapy
Tamoxifen citrate	Estrogen receptor antagonist	Other
Teniposide	Topoisomerase II inhibitor	Chemotherapy
Thalidomide	Immunosuppressant	Other
Thioguanine	Antimetabolite; Purine analog	Chemotherapy
Thiotepa	Alkylating agent	Chemotherapy

Uracil mustard	Alkylating agent	Chemotherapy
Valrubicin	Topoisomerase II inhibitor	Chemotherapy
Vandetanib	VEGFR, EGFR, RET inhibitor	Targeted therapy
Vinblastine sulfate	Mitotic inhibitor. Vinca alkaloid microtubule depolymerizer	Chemotherapy
Vincristine sulfate	Mitotic inhibitor. Vinca alkaloid microtubule depolymerizer	Chemotherapy
Vinorelbine tartrate	Mitotic inhibitor. Vinca alkaloid microtubule depolymerizer	Chemotherapy
Vismodegib	Smothened (Hh) inhibitor	Targeted therapy

Table S2. Performance metrics of the model on original and modified shuffled datasets for O'Neil, ALMANAC, and AZ-Dream.

Dataset	Metric	Original Data	Modified Data
O'Neil	MAE	2.21 ± 0.02	2.20 ± 0.02
	MSE	6.27 ± 0.13	6.27 ± 0.12
	Accuracy	30.42 ± 1.14	30.46 ± 1.09
ALMANAC	MAE	3.07 ± 0.003	3.06 ± 0.003
	MSE	12.26 ± 0.20	12.25 ± 0.18
	Accuracy	38.6 ± 1.12	38.7 ± 0.008
AZ-Dream	MAE	2.67 ± 0.05	2.67 ± 0.06
	MSE	9.51 ± 0.32	9.50 ± 0.30
	Accuracy	20.8 ± 1.07	20.9 ± 1.05
